# Depression among epileptic patients and its association with drug therapy in sub-Saharan Africa: A systematic review and meta-analysis

**DOI:** 10.1371/journal.pone.0202613

**Published:** 2019-03-14

**Authors:** Getenet Dessie, Henok Mulugeta, Cheru Tesema Leshargie, Fasil Wagnew, Sahai Burrowes

**Affiliations:** 1 Department of Nursing, school of health science, College of Medicine and Health Science, Bahir Dar University, Bahir Dar, Ethiopia; 2 Department of Environmental Health, College of Health Science, Debre Markos University, Debre Markos, Ethiopia; 3 Department of Nursing, College of Health Science, Debre Markos University, Debre Markos, Ethiopia; 4 Public Health Program, College of Education and Health Sciences, Touro University California, Vallejo, United States of America; University of Mississippi Medical Center, UNITED STATES

## Abstract

**Background:**

Despite the high prevalence of epilepsy in sub-Saharan Africa and the established relationship between depression and epilepsy, the extent of comorbid epilepsy and depression in the region is still poorly understood. The objective of this systematic review and meta-analysis is to address this gap in the literature by determining the pooled prevalence of depression among epileptic patients in sub-Saharan Africa.

**Methods:**

A systematic desk review and electronic web-based search of PubMed, Google Scholar, EMBASE, PsycINFO and the World Health Organization’s Hinari portal (which includes the SCOPUS, African Index Medicus, and African Journals Online databases) conducted from December 2, 2017 to February 30, 2018, identified peer-reviewed, original research articles and doctoral dissertations using pre-defined quality and inclusion criteria. Relevant data were extracted and descriptive summaries of the studies presented in tabular form. The I^2^ statistic was used to assess heterogeneity across studies. Funnel plot asymmetry and Egger’s tests were used to check for publication bias and the methodological quality of the included studies were assessed using the scale developed by Hoy and colleagues. The pooled prevalence of comorbidity at a 95% confidence interval (CI) was determined by applying a trim and fill analysis in a random-effects model.

**Results:**

Our search identified 167 studies, of which 14 original research articles and two doctoral dissertations reporting on case-control and cross-sectional studies were eligible for inclusion in the final analysis. The pooled estimate of prevalence of depression among patients with epilepsy was 32.71% (95% CI: 25.50–39.91%). Regional sub-group analysis found that the pooled prevalence in East Africa was 34.52% (95% CI: 23.53–45.51%) and 29.69% (95% CI: 22.7–36.68%) in Southern and West Africa. The odds of depression among epileptic patients receiving polytherapy were 2.65 higher than in those receiving monotherapy (95% CI: 1.49–4.71, I^2^ = 79.1%, p < 0.05).

**Conclusion:**

Our findings indicate high comorbidity in sub-Saharan Africa and suggest that it may be more prevalent there than elsewhere. Comorbidity is statistically associated with polytherapy in the studies reviewed. Given the high levels of comorbidity in the region, more attention should be paid to incorporating depression screening and treatment into existing epilepsy programs and to revising treatment guidelines on comorbid depression to reduce polytherapy.

## Introduction

Epilepsy is one of the most common neurological disorders globally, and in sub-Saharan Africa, where it affects approximately 10 million people annually [1]. Untreated, epilepsy can cause traumatic injuries, impair physical functioning, and reduce social engagement. This in turn can result in significant psychological stress and premature death.

People with epilepsy (PWE) are more vulnerable to psychiatric illnesses: rates of psychiatric illness are 9% higher among PWE than in the general population and rates of depression, 22% higher [2]. Depression is the most common psychiatric disorder in PWE [3], and major depressive episodes, one of the most common diagnoses. Social stigma, feelings of frustration and low self-esteem due to the danger and unpredictability of epilepsy, and the psychotropic effects of antiepileptic drugs (AEDs) have all been posited as reasons for the strong association between the two illnesses [4]. Scholars such as Andres Kanner have also argued that the association between depression and epilepsy may be bi-directional, with people who are depressed having higher risk for developing epilepsy perhaps due to “structural, neuropathological, and neurotransmitter disturbances associated with primary major depressive disorders” [5].

Having a depression-related comorbidity is associated with poorer quality of life and increased suicidal ideation for epileptic patients [2,6]. Greater severity of comorbid depression with epilepsy is associated with significantly reduced overall seizure recovery, higher seizure severity, and increased cognitive, emotional, and physical illness [7,8]. Clinicians may also find managing anti-depression treatment particularly challenging for their patients with epilepsy due to concerns about drug interactions, the side effects of polytherapy, and fears of lowering seizure thresholds [9,10].

In addition to complicating treatment and reducing quality of life, depression in PWE also strains health systems, particularly in low-income countries, because PWE with untreated depression tend to use significantly more health resources. For example, Cramer et al.’s studies of health care utilization among comorbid patients found that epileptic patients with mild to moderate depression had a two-fold increase in medical visits, and those with severe depression, a four-fold increase, compared to those who were not depressed [7,11]. Moreover, studies suggest that depressed patients might be less adherent to epilepsy treatment than their non-depressed counterparts [12] and may respond relatively poorly to drug treatment [13].

Several factors have been found to be associated with increased risk of depression in the epileptic population. In sub-Saharan Africa, lower educational status, lower monthly income, frequency of seizure, the side effects of AEDs, and difficulties adhering to AEDs have all been found to be prominent risk factors for depression [14–18]. Polytherapy has also been reported as an important factor, but the association between depression and polypharmacy exhibits significant variation across studies; some finding a significant positive association [14–16,19] and others none [17].

### Prevalence of depression and epilepsy in sub-Saharan Africa

Studying the psychiatric comorbidities of epilepsy in sub-Saharan Africa is important because of the high prevalence of epilepsy in the region. This elevated prevalence is thought to be due to the endemicity of bacterial and parasitic infections that affect the central nervous system and to poor labor and delivery and perinatal care practices that result in head trauma in infants and young children [20,21] For example, febrile convulsions in children brought on by malaria, bronchopneumonia, and upper respiratory tract infections are reported extremely frequently in sub-Saharan African health systems as a significant cause of seizures [1] and it is unclear how often of these febrile convulsions continue as partial epilepsy. In addition, infections such as meningitis, encephalitis, and septicemia might affect the brain directly, leading to epilepsy[1]

Sub-Saharan Africa also has a significant burden of depression. Globally, depressive disorders are the single largest contributor to non-fatal poor health (7.5% of all years lived with disability) and more than 80% of this burden is concentrated in low- and middle-income countries, with Sub-Saharan Africa’s 29 million cases accounting for 9% of the global burden[22]. WHO estimates that 6% of women and 5% of men in sub-Saharan Africa have depression; and with growing populations, urbanization, and aging, it is expected that this prevalence will increase markedly in the coming decades[22]. Despite the significant prevalence of depression in sub-Saharan Africa it, and other behavioral illnesses are rarely prioritized in public health policies[23,24].

It is reasonable to expect that the prevalence of depression and epilepsy comorbidity and its negative health and socio-economic effects would be more pronounced in the sub-Saharan African region where social stigma surrounding epilepsy is pronounced, and the availability of adequate treatment lacking. People with epilepsy in sub-Saharan Africa may experience severe isolation and discrimination in many areas of life, including the health care sector because epilepsy is often perceived as a curse, a mental illness, or a contagious disease[1].

Studying the extent of depression and epilepsy comorbidity in sub-Saharan Africa is complicated by the fact that only about 20% of PWE in low- and middle-income countries receive treatment [1] and by poor estimates of the underlying population prevalence of both diseases. It is difficult to gauge the reliability of epidemiological figures on these diseases because of the lack of consistent standards for data collection on them and, in particular, a tendency to conflate epilepsies with generalized convulsive seizures [1].

While there is relatively consistent evidence of high prevalence of comorbidity globally, most systematic reviews to date have included either no African studies [25,26] or only one or two studies from the continent [27] while the African literature on the subject has been characterized by considerable variability, inconsistency, and inconclusive findings.

Better information on the extent of this comorbidity and its association with polytherapy is important for reducing inappropriate treatment of PWE and for improving suicide prevention efforts in this vulnerable population [28]. Documenting the extent of comorbidity may also help to highlight the need for more active mental health policy making in the region. This systematic review and meta-analysis therefore, aims to synthesize evidence on the prevalence of depression among epileptic adults, children, and adolescents and its association with drug therapy in sub-Saharan Africa.

## Methods

### Search approach and appraisal of studies

Original research articles and doctoral dissertations reviewed in this meta-analysis were accessed through electronic web-based database searches, desk reviews of doctoral dissertations, and reference list reviews using the Preferred Reporting Items of Systematic Reviews and Meta-Analysis (PRISMA) checklist guidelines [29].

The electronic databases searched were PubMed, Google Scholar, Embase, PsycINFO and a World Health Organization (WHO) database portal for low- and middle-income countries that includes the Web of Science, SCOPUS, African Index Medicus (AIM), Cumulative Index to Nursing and Allied Health Literature (CINAHL), WHO’s Institutional Repository for Information Sharing (IRIS) and African Journals Online databases. In addition, the researchers found related articles through a desk review of the doctoral dissertations available at Ethiopian university libraries and institutional repositories, and from reviewing the reference lists of related articles.

Searches were conducted from December 1, 2017 to January 30, 2018. The researchers used, among others, the following key terms for the database searches: “depression” AND “epilepsy” OR “co-morbid depression” AND “epilepsy” OR “mental illness” AND drug therapy “OR “treatment” AND “sub-Saharan Africa*”*. Please see the S1 Table for a list of exact search terms used for each database and the number of results found in each search.

### Inclusion and exclusion criteria

All English-language, full-text, original research articles and doctoral dissertations on observational studies (case-control or cross-sectional) conducted in the sub-Saharan Africa region from 2005 to 2017, with adults, children or adolescents, that were published in peer-reviewed journals or filed as completed dissertations, that used internationally accepted scales to measure epilepsy and depression (e.g., Beck’s Depression Inventory scale or the Hospital Anxiety and Depression Scale), that defined epilepsy and depression according to internationally accepted definitions (e.g., DSM-IV), and that concerned comorbid depression and epilepsy were eligible for inclusion.

### Data extraction and quality assessment

After initial screening, two reviewers (FW and GD) downloaded abstracts to assess them for inclusion. If reviewers disagreed about whether a search result was relevant to the study, it was included for retrieval. The relevance of the items was then evaluated based on the item’s title, topic, objectives, and methodology as listed in the abstract. Abstracts were also assessed for agreement with the inclusion criteria. At this stage, articles deemed irrelevant or out of the scope of the study were excluded and the full text of the remainder downloaded for a detailed review. When it was unclear whether an abstract was relevant, or there was disagreement among reviewers on whether it met the inclusion criteria, it was included for retrieval. Two reviewers (GD and FW) then assessed the quality of potentially eligible articles using the Newcastle-Ottawa Scale (NOS) criteria [30]. The average of two independent reviewers’ score was used to determine whether the articles should be included. Discrepancies in quality assessment scores were resolved with a third reviewer (HM), whenever appropriate. Articles whose NOS quality scores were less than six; those that had methodological flaws, or incomplete reporting of results; or those for which full text was not available were excluded from the final analysis. Study researchers made two separate attempts to contact article authors whenever additional study information was needed; for example, when patient outcome data were incomplete.

### Outcome of interest

The outcome of interest was the pooled prevalence of depression among epileptic patients in sub-Saharan Africa. Prevalence was measured as the number of comorbid study subjects divided by the number of patients in a study multiplied by 100. We also estimated the association (as measured by crude odds ratios) between comorbidity and polytherapy as a secondary outcome.

### Data analysis

Information on the study characteristics (time frame, study location, study design, sample size, number of comorbid patients, and the age-range of patients) was extracted from each study using a Microsoft Excel spreadsheet template. These data were then transferred to Stata version 11 software, which was used to calculate the pooled prevalence of depression among epileptic patients, perform tests, and to identify the significant association between the outcome variable and factors. The heterogeneity of study outcomes was assessed using the I^2^ statistic [31]. We used funnel plot asymmetry and Egger’s and Begg-Mazumdar Rank correlation tests to check for publication bias [32]. Because the results of these tests suggested the possible existence of significant publication bias, a random effects model was used to estimate the pooled prevalence of comorbidity at a 95% confidence interval (CI) [33]. In addition, we conducted a geographic subgroup analysis. To confirm results, two researchers independently carried out the main statistical analysis and results were cross-checked for consistency.

### Assessment of methodological quality of included studies and risk of bias

We evaluated the risk of bias in the studies that were selected using the 10-item rating scale developed Hoy et al. for prevalence studies (see S2 Table) [34]. The tool assesses studies on 10 domains, including sampling, data collection, reliability and validity of study tools, case definition, and prevalence periods. Researchers categorized each study as having low risk of bias (“yes” answers to domain questions) or high risk of bias ("no" answers to domain questions). Each study was assigned a score of 1 (Yes) or 0 (No) for each domain, and these scores were summed to provide an overall study quality score. Scores of 8–10 were considered as having a “low risk of bias”, 6–7 a “moderate risk”, and 0–5 a “high risk”. For the final risk of bias classification, disagreements between the reviewers were resolved via consensus.

## Results

### Identification and description of studies

**Fig 1:**
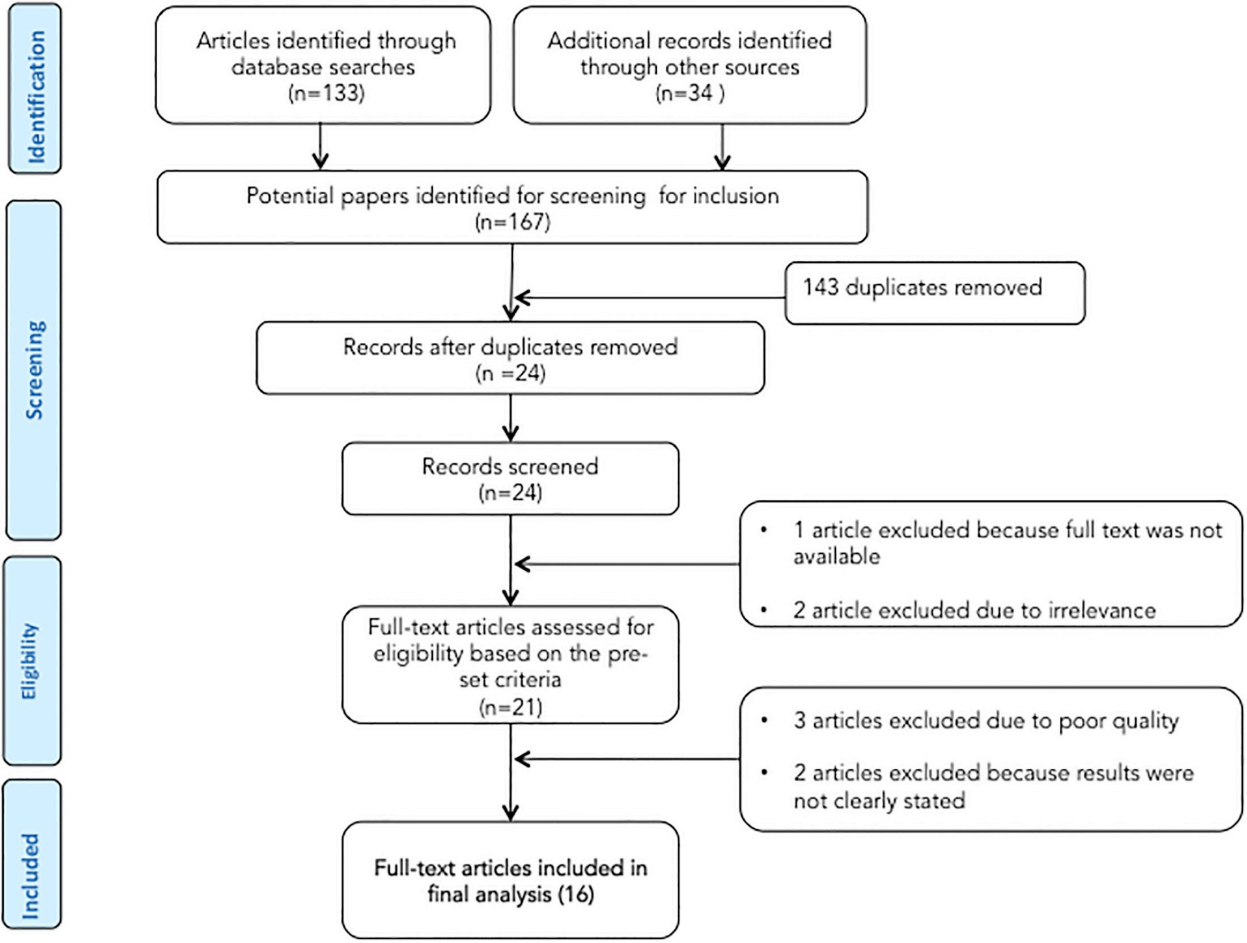
Flow diagram for selection of studies.

The database search and desk review yielded a total of 167 articles (Fig 1). Of these, 133 articles were found in PubMed, EMBASE, PsycINFO, Hinari and Google Scholar and the remaining 34 were found from the desk review. After reviewing the titles and abstracts, we excluded 143 items due to duplication. The abstracts of the remaining 24 items were downloaded and assessed for eligibility. One article from Ethiopia was excluded because the full text was not available [35]. Two articles from Zambia were excluded due to irrelevance [36,37]. In step three, the full text of the remaining 21 articles was assessed for quality and appropriateness. Three articles from Nigeria [38], Togo [39] and Benin [39] were excluded due to low quality scores. Two articles from Sierra Leone [40] and Zambia [41] were excluded because the study outcome was not clearly stated.

The remaining 16 studies were included in the analysis (Fig 1). The 16 articles reported on six cross-sectional studies from Ethiopia [15–18,42,43], one cross-sectional study from Kenya [14], four cross-sectional studies and one case–control study from Nigeria [44–48], two cross-sectional studies from Rwanda [49,50], one case–control study from Sudan [51] and one cross-sectional study from Zambia [52].

### Characteristics of included studies

Sixteen studies with a total sample of 4,314 epileptic patients whose ages ranged from 9 to 65 were assessed (Table 1). The majority of studies were conducted in Ethiopia [15–18,42,43], and Nigeria [44–48]. The remaining were from Rwanda [49,50], Sudan [51], Zambia [52] and Kenya [14]. Studies were mostly found in small, regional, peer-reviewed journals, and two were reported in doctoral dissertations.

**Table 1:** Characteristics of included studies for systematic review and meta-analysis, 2005–2017, sub-Saharan Africa.

Study	Source Type	Study year	Country (Region)	Study design	Age of subjects	Sample size	Response rate	Depression measure	Depression prevalence (%)	Quality Score	Factors positively associated with depression
Tsegabrhan, et al [17]	Journal	2014	Ethiopia (East)	Cross-sectional	≥18 years	300	100%	BDI	49.3	7	Lower educational status Higher seizure frequency Higher perceived stigma
Tegegne et al [16]	Journal	2015	Ethiopia (East)	Cross-sectional	≥18 years	415	98%	HADS	32.8	8	Lower educational status Polytherapy Higher perceived stigma
Bifftu et al [15]	Journal	2015	Ethiopia (East)	Cross-sectional	≥18 years	405	96%	BDI	45.2	7	Lower educational status Higher seizure frequency Polytherapy Higher perceived stigma Early onset of seizures Difficulty adhering to AEDs
Tegegne et al [43]	Journal	2014	Ethiopia (East)	Cross-sectional	≥18 years	415	98%	HADS	32.8	8	(not assessed)
Bifftu et al [42]	Journal	2015	Ethiopia (East)	Cross-sectional	≥24 years	408	97%	BDI-II	45.1	8	(not assessed)
Tilahune et al [18]	Journal	2016	Ethiopia (East)	Cross-sectional	≥18 years	326	100%	PHQ	24.5	7	Female gender Greater age Divorced Lower income
Kiko [14]	Dissertation	2013	Kenya (East)	Cross-sectional	≥18 years	327	Not stated	BDI	16.5	7	Polytherapy *(associated with mild depression)*
Adewuya et al[44]	Journal	2005	Nigeria (West)	Cross-sectional	12–18 years	102	90%	DISC-IV	28.4	8	Polytherapy Higher perceived stigma Uncontrolled seizures
Ayanda et al [45]	Journal	2016	Nigeria (West)	Cross-sectional	18–68 years	74	Not stated	MINI	21.6	8	Not assessed
Owolabi et al [48]	Journal	2016	Nigeria (West)	Cross-sectional	≥18 years	255	Not stated	MINI	20.4	8	Higher seizure frequency Early onset of seizures (duration of epilepsy) Previous hospitalization for epilepsy
Mosaku et al [46]	Journal	2006	Nigeria (West)	Cross-sectional	18-38years	51	Not stated	HADS	27.5	7	Not assessed
Ogunrin et al [47]	Journal	2010	Nigeria (West)	Case–control	18-65years	152	Not stated	BDI, HRSD	42	8	Female gender Uncontrolled seizures Duration of epilepsy Difficulty adhering to AEDs *(Associated with both BDI & HRSD)*
Sezibera et al [50]	Journal	2013	Rwanda (East)	Cross-sectional	9–68 years	105	Not stated	HRSD	48.6	8	Female gender Greater age Lower educational status
Mutabazi [49]	Dissertation	2014	Rwanda (East)	Cross-sectional	18–73 years	382	Not stated	MINI	6.5	8	Not assessed
Saadalla et al [51]	Journal	2016	Sudan (East)	Case–control	18–70 years	200	Not stated	BDI	45.5	7	Not assessed
Veneviv et al [52]	Journal	2016	Zambia (South)	Cross-sectional	≥18 years	397	Not stated	BPRS	39.4	7	Not assessed

Beck’s Depression Inventory (BDI); Brief Psychiatric Rating Scale (BPRS); Diagnostic Interview Schedule for Children Version IV (DISC-IV); Hospital Anxiety and Depression Scale (HADS); Hamilton Rating Scale for Depression (HRSD); Mini International Neuropsychiatric Interview (MINI); Patient Health Questionnaire.

Most studies were cross-sectional, but two [47,51] used a case-control design. All studies recruited participants from in-patient or out-patient clinical settings. Five of the studies stated the age of onset of epilepsy and its duration. In these studies, the age of epilepsy onset ranged from 9 to 30 years of age [15,16,42,43,48] and the duration of disease ranged from 1 to 15 years [15,16,42,43,52]. Half of the studies examined the factors associated with depression in PWE; four studies examined the factors related to PWE having depressive disorder [15,16,43,47].

### Quality assessment and risk of bias

Most studies had moderate sample sizes; only two had small samples of less than 100 participants [45,46]. Reported response rates were high (>90%) but more than half of the studies did not report a response rate and only one discussed the characteristics of non-responders. All studies used standardized methods for measuring depression with the Beck’s Depression Inventory (BDI) and the Hospital Anxiety and Depression Scale (HADS) being the tools most frequently used.

Due to our initial quality screening, all of the studies included in the final review had either medium or high quality using the Hoy et al. risk of bias [34] tool. Twelve of the 16 studies (75%) received a high quality score (≥8 points) and the remaining 4 studies (25%) were scored as being of medium quality (5–7 points) (see S2 Table).

### Publication bias

**Fig 2:**
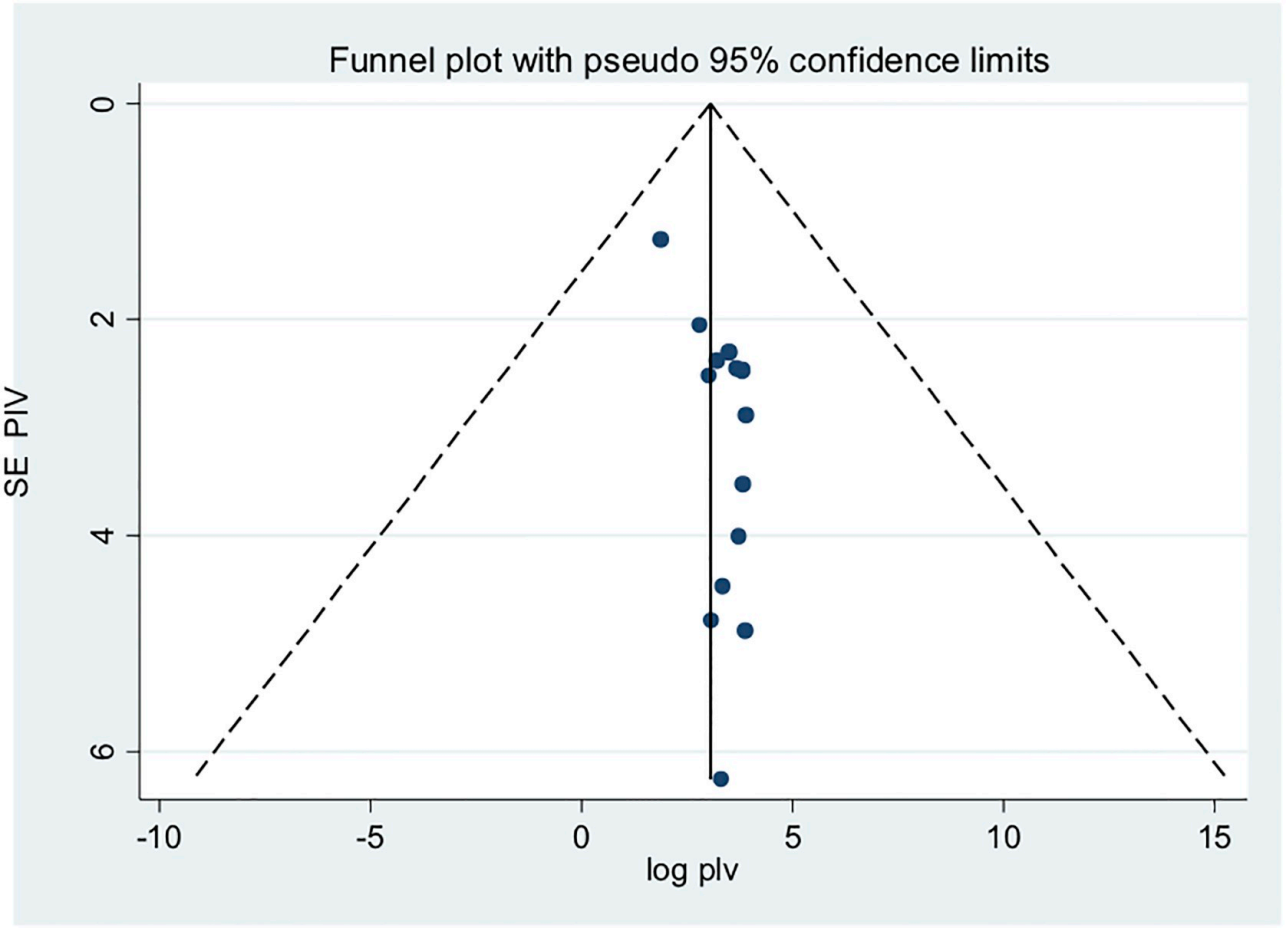
Funnel plot for systematic review of the prevalence of depression among epileptic patients, 2005–2017 in sub-Saharan Africa.

Both funnel plots of precision asymmetry and the Egger’s test of the intercept indicated the presence of publication bias. Visual examination of the funnel plot showed it to be asymmetric (Fig 2) and Egger’s test of the intercept (B0) was 0.54 (95% CI: 0.20–0.87 p<0.05). To mitigate against publication bias we applied a trim and fill analysis in the random effects model. The prevalence estimates did not differ significantly between the initial model and the trim and fill model.

### Prevalence of depression in PWE

Depression prevalence ranged from a high of 49.3% in an Ethiopian study [17] to a low of 6.5%, reported in a study conducted in Rwanda [49].

**Fig 3:**
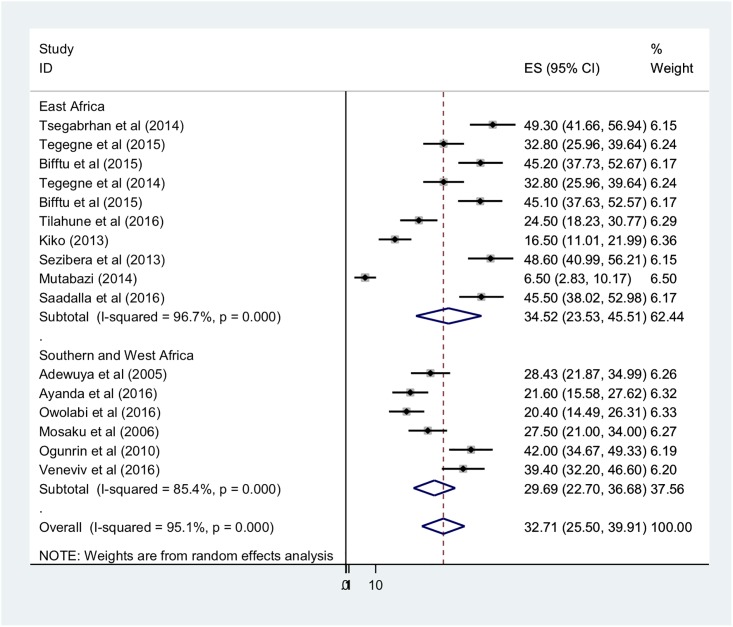
Forest plot of 16 studies assessing prevalence of depression among epilepsy patients, 2005–2017, sub-Saharan Africa.

Because the I^2^ statistic for heterogeneity indicated significant difference between the studies (I^2^ = 98%, p<0.05) and because theoretically we expected that the settings and socio-economic contexts might differ radically across these studies, we fitted a DerSimonian and Laird random effects model to estimate the pooled prevalence of depression [53,54]. In the model, each individual study is given a weight based on its reported effect size and sample size [55]. The studies with the largest weight were Michel [49], Kiko [14], and Tegegne et al [16] with respective weights of 6.5%, 6.4%, and 6.4%. Smaller weights were given for Mosaku, 5.7%, [46], Sezibera et al 6.0%, [50] and Ayanda et al 6.0% [45] (Fig 3).

The average pooled estimate of depression among epilepsy patients was 32.71% (95% CI: 25.50–39.91%) (Fig 3). Sub-group analysis by geographic region found that the pooled prevalence of depression among epileptic patients in East Africa was 34.52% (95% CI: 23.53–45.51%) and among patients in Southern and West Africa, 29.69% (95% CI: 22.7–36.68%) (Fig 3).

### Factors associated with depression among PWE

The factors most frequently associated with depression in PWE were, in order, lower educational status [15–17,50], higher perceived stigma [15–17,44], polytherapy [14,16,17,44], female gender [18,47,50], the frequency of seizures [15,17,48], having controlled seizures [44,47], the duration of epilepsy [15,47] and greater age [18,50].

**Fig 4:**
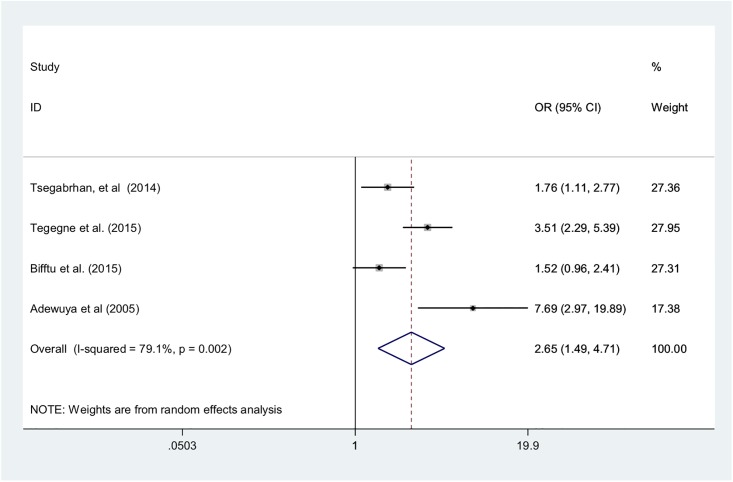
Forest plot of four studies examining the association of polytherapy with depression among epilepsy patients, 2005–2017, sub-Saharan Africa.

Four of the studies explicitly intended to find factors associated with depression in epilepsy patients [15–17,44]. One of the factors most strongly associated with depression among PWE in these studies was polytherapy (i.e., the amount of different medications patients received). The pooled odds of depression among epilepsy patients receiving polytherapy was 2.65 (95% CI: 1.49–4.71) when compared with patients receiving monotherapy (Fig 4).

## Discussion

### Key findings

This systemic review and meta-analysis attempted to estimate the pooled prevalence of depression and its association with polytherapy among PWE in sub-Saharan Africa. We found a very high prevalence of depression (32.71%) among PWE. This estimate was roughly 10 percentage points larger than that reported in recent meta-analyses, which reviewed studies that were almost exclusively conducted in Europe, the Americas, or Asia [25–27]. In the one recent cross-national meta-analysis that included two studies from sub-Saharan Africa, the region was found to have the highest regional prevalence of comorbidity, although that pooled prevalence, 25.6%, was lower than that found in our study [27].

Although we expected to find significant comorbidity due to the well-established bi-directional relationship between the depression and epilepsy [3,56], the high prevalence of depression reported in the studies under review suggest that the psychological toll of the epilepsy may be particularly severe in sub-Saharan African settings where the illness is poorly managed, treatment options limited, epilepsy-related stigma high, and the social and economic costs of illness particularly acute. The relationship between depression and epilepsy is not only neurological but also triggered and shaped by socioeconomic factors [57]. In settings where epilepsy is thought to be a potentially contagious spiritual curse, PWE face profound social isolation and limited prospects for education, employment, and marriage [21] leaving them physically, emotionally, and economically vulnerable. In addition, endemic poverty in many communities means that PWE must manage the stress of affording treatment while maintaining their livelihoods in households that are often food insecure and financially precarious. The argument that high social stigma, poor treatment, and low socio-economic status in sub-Saharan Africa are important drivers of the region’s higher rates of comorbidity is supported by the findings in the individual studies reviewed that these factors are significantly associated with depression among PWE.

A secondary objective of this study was to determine the effect of polytherapy on the prevalence of depression in PWE. We found that polytherapy doubled the odds of depression (OR = 2.65; 95% CI: 1.49–4.71) compared with monotherapy. Polytherapy not only increases pill burden, making adherence, and therefore control of seizures, more difficult, it also increases the risk of adverse drug reactions and the severity of those reactions [58,59]. In addition, patients taking multiple medications have higher risk of drug-to-drug interactions and may be more prone to medication errors [58,59]. Our findings suggest that greater emphasis should be placed on assessing pill burden, tolerability, and potential drug interactions as well as on providing appropriate health education about drug regimens when designing epilepsy treatment guidelines and health care provider training curricula.

Our regional sub-group analysis found higher prevalence of depression in East Africa than in Southern and West Africa (34.52% vs. 29.69%). This difference may be due to the large number of East African studies that were conducted in Ethiopia, which has a particularly weak mental health care system compared to other regions. Poorly controlled epilepsy is a risk factor for depression and Ethiopia lacks the person power and infrastructure to manage epilepsy well for the majority of patients in need. For example, for a country of almost 100 million people, Ethiopia has only approximately 63 psychiatrists, 150 Masters-level mental health professionals, and 200 Bachelors-level psychiatry nurses [60] and most of these providers are concentrated in the capital city. Non-mental health professionals are often assigned to provide mental health services in Ethiopia, but studies indicate that they lack the knowledge required to deliver comprehensive mental health care [61]. Ethiopia’s mental health system also faces many other challenges that complicate treatment of epilepsy such as delayed and inadequate supportive supervisions for trainees, lack of funding for supportive supervision and mentoring, and interrupted drug supplies [60]. Ethiopia’s overall level poverty compared to Zambia and Nigeria may also contribute to the difference in regional prevalence rates, since as discussed above, poor socio-economic status is associated with depression in PWE.

### Study limitations

There are several limitations in this review. First, we were only able to review English-language studies because we lacked investigators who were fluent in the other languages of interest (French and Portuguese), and we were reluctant to introduce translation biases by using translation services to search for, and to translate articles. This may have caused us to omit important studies from Francophone and Lusophone Africa and, therefore, may have reduced the credibility of this review. However, it has been found that language bias in meta-analyses reporting pooled estimates, as this study does, may be less severe than in other types of reviews [62,63]. Our second limitation is that many of the studies reviewed were published in small regional journals or were doctoral dissertations making it difficult to gauge the extent of peer review. Moreover, due to the absence of data, crude odds ratios were used to estimate factors related with comorbidity, which prevented us excluding confounding factors. Finally, our systematic review and meta-analysis protocol was not registered online. The findings of this meta-analysis would be best interpreted keeping these analytical limitations and the limitations of the original studies in mind.

## Conclusion

This meta-analysis found that the prevalence of comorbid depression with epilepsy in sub-Saharan Africa was high, and may be of greater magnitude than that reported in other geographic regions. We also find that in these studies, comorbidity is significantly associated with polytherapy. These findings together with the high overall prevalence of epilepsy in sub-Saharan Africa, suggest that more attention should be paid to increasing health education on epilepsy in order to reduce stigma. Incorporating depression screening and treatment into existing epilepsy programs and revising treatment guidelines on comorbid depression to reduce polytherapy may also be warranted. Future research on this subject in sub-Saharan Africa should focus on identifying appropriate medication regimens for patients with comorbid depression.

## Supporting information

S1 Table Search strategy. (PDF)

S2 Table Tables for assessment of methodological quality of included studies and risk of bias. (PDF)

S3 Table PRISMA checklist. (DOCX)
