# Supplementary material for: Depression among epileptic patients and its association with drug therapy in sub-Saharan Africa: A systematic review and meta-analysis
Source: PLoS One. 2019 Mar 14;14(3):e0202613. doi: 10.1371/journal.pone.0202613 (PMC6417665; doi:10.1371/journal.pone.0202613)
Supplement: S1 Table — (PDF) [file pone.0202613.s001.pdf]

Table 3: Search Strategy

Databases	Search Terms	No of articles identified
**PubMed**	("depressive disorder"[MeSH Terms] OR ("depressive"[All Fields] AND "disorder"[All Fields]) OR "depressive disorder"[All Fields] OR "depression"[All Fields] OR "depression"[MeSH Terms]) AND ("epilepsy"[MeSH Terms] OR "epilepsy"[All Fields] OR "epileptic"[All Fields]) AND ("patients"[MeSH Terms] OR "patients"[All Fields]) AND Associated[All Fields] AND factors[All Fields] AND ("africa south of the sahara"[MeSH Terms] OR ("africa"[All Fields] AND "south"[All Fields] AND "sahara"[All Fields]) OR "africa south of the sahara"[All Fields] OR ("sub"[All Fields] AND "saharan"[All Fields] AND "africa"[All Fields]) OR "sub Saharan africa"[All Fields])	41
**CINAHL (Plus with full text)**	**Thesaurus terms:** depression, drug therapy, epilepsy, prevalence, sub-Saharan Africa<br>**Search Terms:** " depression disorder *" OR depressive * OR " depression *" and " epilepsy *" OR " epileptic " AND " drug therapy "OR" treatment" AND "Africa OR sub-Saharan Africa<br>(S1): filter: English, peer reviewed, exclude Medline<br>(S2): with additional filter: Africa, sub-Saharan Africa<br>S1 OR S2	22
**Cochrane Library**	**MeSH terms:** depression disorder (MeSH), depressive (MeSH), depression (MeSH), epilepsy (MeSH), epileptic (MeSH), Associated (MeSH) factors (MeSH).<br>**Search Terms:** (depression disorder * OR depressive * OR depression * AND (epilepsy[MeSH] OR epileptic[MeSH] AND drug therapy AND sub-Saharan Africa AND English [la]	21
**PsycINFO**	**Thesaurus terms:** depression, drug therapy, epilepsy, prevalence, sub-Saharan Africa<br>**Search Terms:**<br>" depression disorder *" OR depressive * OR " depression " and "epilepsy*" OR "epileptic" AND drug therapy OR treatment* AND sub-Saharan Africa<br>filter: English, peer reviewed	33
**Embase**	**Emtree terms:** depression, drug therapy, epilepsy, sub-Saharan Africa<br>**Search 1 Terms:** (depression disorder * OR depressive OR depression* AND (epilepsy OR epileptic * AND drug therapy AND (embase) NOT (medline)<br>**Search 2 Terms:** (depression disorder NEXT/1 depressive * OR depression * NEXT/1 depressive * OR depression NEXT/1 phone* AND (epilepsy OR epileptic) AND (drug therapy OR treatment) AND english:la AND [embase]/lim NOT [medline]/lim<br>filter English only and excluded Medline	16
**Grey Literature; Google & Google Scholar**	Variety of key terms used from the above searches	34
