# Supplementary material for: Depression among epileptic patients and its association with drug therapy in sub-Saharan Africa: A systematic review and meta-analysis
Source: PLoS One. 2019 Mar 14;14(3):e0202613. doi: 10.1371/journal.pone.0202613 (PMC6417665; doi:10.1371/journal.pone.0202613)
Supplement: S2 Table — (PDF) [file pone.0202613.s002.pdf]

	Study ID	Representat ion	Sampling	Random selection	Non- response bias	Data collection	Case definition	Reliability & validity	Method of data collection	Prevalence period	Numerator & denominator	Summary assessment
1	Tsegabrhan et al	0	1	0	1	1	0	0	1	1	1	**6**
2	Tegegne et al	0	1	1	0	1	1	1	1	1	1	**8**
3	Bifftu et al	1	1	0	0	1	1	0	1	1	1	**6**
4	Tegegne et al	1	1	0	1	0	0	0	1	1	1	**6**
5	Bifftu et al	0	1	1	1	1	1	1	1	1	1	**9**
6	Tilahune et al	0	1	0	1	1	1	1	1	1	1	**8**
7	Kiko	0	1	1	1	1	1	1	1	1	1	**9**
8	Adewuya et al	0	1	1	1	1	1	1	1	1	1	**9**
9	Ayanda et al	0	0	1	1	1	1	1	1	1	1	**8**
10	Owolabi et al	1	1	1	1	1	1	1	1	1	1	**10**
11	Mosaku et al	0	1	1	1	1	0	1	1	1	1	**8**
12	Ogunrin et al	0	1	1	0	1	1	0	1	1	1	**7**
13	Sezibera et al	0	1	1	0	1	1	1	1	1	1	**8**
14	Mutabazi	1	1	1	1	1	1	1	1	1	1	**10**
15	Saadalla et al	1	1	1	1	1	0	1	1	1	1	**9**
16	Veneviv et al	1	1	1	1	1	0	1	1	1	1	**9**

Yes=0, No=1

The overall risk of bias scored based on the number of high risk of bias per study: 8–10: 'low risk of bias', 5–7: 'moderate risk' and 0–4: 'high risk'.

Risk of bias assessment tool: Yes (low risk); No (high risk)

1. Representation: Was the study population a close representation of the national population?
2. Sampling: Was the sampling frame a true or close representation of the target population?
3. Random selection: Was some form of random selection used to select the sample OR was a census undertaken?
4. Non-response bias: Was the likelihood of non-response bias minimal?
5. Data collection: Were data collected directly from the subjects?
6. Case definition: Was an acceptable case definition used in the study?
7. Reliability and validity of study tool: Was the study instrument that measured the parameter of interest show to have reliability and validity?
8. Data collection: Was the same mode of data collection used for all subjects?
9. Prevalence period: Was the length of the prevalence period for the parameter of interest appropriate?
10. Numerators and denominators: Were the numerator(s) and denominator(s) for the parameter of interest appropriate?
