# Supplementary material for: Depression among epileptic patients and its association with drug therapy in sub-Saharan Africa: A systematic review and meta-analysis
Source: PLoS One. 2019 Mar 14;14(3):e0202613. doi: 10.1371/journal.pone.0202613 (PMC6417665; doi:10.1371/journal.pone.0202613)
Supplement: S3 Table — (DOCX) [file pone.0202613.s003.docx]

| **Section/topic** | **#** | PRISMA  **Checklist item** | **Reported on page #** |
| --- | --- | --- | --- |
| **TITLE** | | | 1 |
| Title | 1 | **Prevalence of Depression among Epileptic Patients and its Association with Drug Therapy: A Systematic Review and Meta-Analysis** |  |
| **ABSTRACT** | | | 2-3 |
| Structured summary | 2 | **Abstract**  **Background:** Despite the high prevalence of epilepsy in sub-Saharan Africa and the established relationship between depression and epilepsy, the extent of comorbid epilepsy and depression in the region is still poorly understood. The objective of this systematic review and meta-analysis is to address this gap in the literature by determining the pooled prevalence of depression among epileptic patients in sub-Saharan Africa.  **Methods:** A systematic desk review and electronic web-based search of PubMed, Google Scholar, EMBASE, PsycINFO and the World Health Organization’s Hinari portal (which includes the SCOPUS, African Index Medicus, and African Journals Online databases) conducted from December 2, 2017 to February 30, 2018, identified peer-reviewed, original research articles and doctoral dissertations using pre-defined quality and inclusion criteria. Relevant data were extracted and descriptive summaries of the studies presented in tabular form. The I^2^ statistic was used to assess heterogeneity across studies. Funnel plot asymmetry and Egger’s tests were used to check for publication bias and the methodological quality of the included studies were assessed using the scale developed by Hoy and colleagues. The pooled prevalence of comorbidity at a 95% confidence interval (CI) was determined by applying a trim and fill analysis in a random-effects model.  **Results:** Our search identified 167 studies, of which 14 original research articles and two doctoral dissertations reporting on case-control and cross-sectional studies were eligible for inclusion in the final analysis. The pooled estimate of prevalence of depression among patients with epilepsy was 32.71% (95% CI: 25.50 - 39.91%). Regional sub-group analysis found that the pooled prevalence in East Africa was 34.52% (95% CI: 23.53 - 45.51%) and 29.69% (95% CI: 22.7 - 36.68%) in Southern and West Africa. The odds of depression among epileptic patients receiving polytherapy were 2.65 higher than in those receiving monotherapy (95% CI: 1.49 - 4.71, I^2^=79.1%, p < 0.05).  **Conclusion:** Our findings indicate high comorbidity in sub-Saharan Africa and suggest that it may be more prevalent there than elsewhere. Comorbidity is statistically associated with polytherapy in the studies reviewed. Given the high levels of comorbidity in the region, more attention should be paid to incorporating depression screening and treatment into existing epilepsy programs and to revising treatment guidelines on comorbid depression to reduce polytherapy. | 2 |
| **INTRODUCTION** | | | 3-7 |
| Rationale | 3 | It is also reasonable to expect that the prevalence of depression comorbidity and its negative health and socio-economic effects would be more pronounced in the sub-Saharan African region where social stigma surrounding epilepsy is more pronounced, and the availability of adequate treatment lacking.However, the evidence base still remains inconsistence and inconclusive. | 7 |
| Objectives | 4 | This systematic review and meta-analysis is therefore aimed to summarize the prevalence of depression among epileptic adults and adolescents and its association with drug therapy in Sub-Saharan Africa. | 7 |
| **METHODS** | | | 7-11 |
| Protocol and registration | 5 | There is no registration number. |  |
| Eligibility criteria | 6 | All English-language, full-text articles on observational studies (case-control or cross sectional) conducted in the sub-Saharan Africa region from 2005 to 2017. | 8 |
| Information sources | 7 | PubMed, Google Scholar, embase, PsycINFO and a World Health Organization (WHO) database portal for low- and middle-income countries that includes the Web of Science, SCOPUS, African Index Medicus (AIM), Cumulative Index to Nursing and Allied Health Literature (CINAHL), WHO’s Institutional Repository for Information Sharing (IRIS) and African Journals Online databases. | 8 |
| Search | 8 | *“*depression*” AND “*epilepsy*” OR “*co-morbid depression*” AND “*epilepsy*” OR “mental illness” AND “*sub-Saharan Africa*”.* | 8 |
| Study selection | 9 | Both published and unpublished literature to estimate the prevalence of depression among epilepsy patients | 9 |
| Data collection process | 10 | Data from the included studies were extracted independently by these reviewers(GD&FW). | 9 |
| Data items | 11 | Data on author(s), study year, region of study, study design ,sample size and factors | 9 |
| Risk of bias in individual studies | 12 | Newcastle-Ottawa Scale adapted for cross-sectional studies | 9 |
| Summary measures | 13 | Prevalence, OR | 9 |
| Synthesis of results | 14 | Subgroup analyses by regions were done due to significance heterogeneity. | 9 |

Page 1 of 2

| **Section/topic** | **#** | **Checklist item** | **Reported on page #** |
| --- | --- | --- | --- |
| Risk of bias across studies | 15 | Begg’s, Egger’s tests, and trim-and-fill analysis was considered | 10 |
| Additional analyses | 16 | Subgroup analyses by region | 9 |
| **RESULTS** | | | 11-16 |
| Study selection | 17 | After reviewing the titles and abstracts we excluded 143 articles due to irrelevance. The full text of the remaining 24 articles were downloaded and assessed for quality and relevance and 16 studies which fulfilled the eligibility criteria, were included in the final meta-analysis | 11 |
| Study characteristics | 18 | Data on author(s), study year, region of study, study design , sample size and factors | 12-14 |
| Risk of bias within studies | 19 | Present data on risk of bias of each study and, if available, any outcome level assessment. | 14 |
| Results of individual studies | 20 | Result of individual studies were presented | 15 |
| Synthesis of results | 21 | Subgroup analysis: in East Africa was 34.52 (95% CI: 23.53 - 45.51) and 29.69 (95% CI: 22.7 - 36.68) among patients in Southern and West Africa | 16 |
| Risk of bias across studies | 22 | Present results of any assessment of risk of bias across studies. | 14 |
| Additional analysis | 23 | Give results of additional analyses, if done (e.g., subgroup analyses) | 16 |
| **DISCUSSION** | | | 16-19 |
| Summary of evidence | 24 | This meta-analysis found that the prevalence of comorbid depression with epilepsy in sub-Saharan Africa was high, and of greater magnitude than that reported in other geographic regions. More attention should be paid to increasing health education on epilepsy to reduce stigma; to incorporating depression screening and treatment into existing epilepsy programs; and to revising treatment guidelines on co-morbid depression to reduce polytherapy. | 19 |
| Limitations | 25 | The inclusion of studies published only in English may compromise representativeness (language bias). | 18 |
| Conclusions | 26 | Depression is a common co-morbidity in epilepsy patients. the co-morbidity is significantly higher in patients with polytherapy | 19 |
| **FUNDING** | | | 20 |
| Funding | 27 | No | 20 |

*From:*  Moher D, Liberati A, Tetzlaff J, Altman DG, The PRISMA Group (2009). Preferred Reporting Items for Systematic Reviews and Meta-Analyses: The PRISMA Statement. PLoS Med 6(7): e1000097. doi:10.1371/journal.pmed1000097

For more information, visit: **www.prisma-statement.org**.

Page 2 of 2
